# Association between gender-based discrimination and medical visits and HIV testing in a large sample of transgender women in northeast Brazil

**DOI:** 10.1186/s12939-021-01541-z

**Published:** 2021-09-06

**Authors:** Beo Oliveira Leite, Danielle Souto de Medeiros, Laio Magno, Francisco Inácio Bastos, Carolina Coutinho, Ana Maria de Brito, Maria Socorro Cavalcante, Inês Dourado

**Affiliations:** 1 Multidisciplinary Health Institute, Federal University of Bahia, Vitória da Conquista, Bahia, Brazil; 2 Collective Health Institute, Federal University of Bahia. Av. Basílio da Gama, s/nCampus Universitário do Canela, Salvador, Bahia 40110-040 Brazil; 3 Life Sciences Department, Bahia State University, Campus1, Salvador, Bahia Brazil; 4 Oswaldo Cruz Foundation, Rio de Janeiro, Rio de Janeiro Brazil; 5 Getúlio Vargas Foundation, São Paulo, São Paulo Brazil; 6 Aggeu Magalhães Institute. Oswaldo Cruz Foundation, Recife, Brazil; 7 Ceará State Health Secretariat and Municipality of Fortaleza, Fortaleza, Brazil

**Keywords:** Transgender Women, Discrimination, Stigma, Use of health services, HIV testing

## Abstract

**Background:**

Gender-based discrimination remains a substantial barrier to health care access and HIV prevention among transgender women in Brazil. The aim of this study was to investigate the association between gender-based discrimination and medical visits, as well as with HIV testing among transgender women in the last 12 months in northeast Brazil.

**Methods:**

This is a cross-sectional study of 864 transgender women recruited using Respondent-Driven Sampling in three cities in northeastern Brazil in 2016. A socio-behavioral questionnaire was applied. Multivariate analyses were performed using logistic regression, with odds ratio and respective 95% confidence intervals estimation, to estimate the effect of gender-based discrimination on two outcomes: i) medical visits and ii) HIV testing in the last 12 months.

**Results:**

547 transgender women (67·0%) had medical visits, and 385 (45·8%) underwent HIV testing in the last 12 months. In the multivariate analysis, gender-based discrimination was associated with a reduced likelihood of medical visits (OR: 0·29; 95%CI: 0·14–0·63) and HIV testing (OR: 0·41; 95%CI: 0·22–0·78) in the last 12 months.

**Conclusion:**

Gender-based discrimination played an essential role in reducing the access of TGW to medical visits and HIV testing services. Furthermore, by confirming the association between gender-based discrimination and medical visits and HIV testing in the multivariate analysis, we have demonstrated how this predictive variable can affect by reducing access to health services. The findings point to the need for non-discriminatory policies based on the defense and promotion of human rights that may foster the access of transgender women to Brazilian health services.

## Background

Transgender women (TGW) are still disproportionately affected by the HIV epidemic, despite the global decrease in incidence over the years [1]. The worldwide estimate of HIV prevalence in this population is 19.1%, however, in Brazil, this prevalence is 33.1%, with an 84.3 times higher probability of infection than the general population of reproductive age [2].

UNAIDS 90–90-90 target establishes that, by the end of 2020, at least 90% of people living with HIV should know their status, 90% of those diagnosed should start antiretroviral treatment, and 90% of those being treated should achieve viral suppression. One of the best ways to ensure people living with HIV should know their status, start antiretroviral treatment, and being treated should achieve viral suppression, is the implementation of strategies to assure competent health services to approach and care key populations [3, 4].

The health care model geared to people at risk of HIV, suggested by the World Health Organization (WHO), basically consists of a network of services: prevention technologies (condoms, lubricants, and pre- and post-exposure prophylaxis), regular testing services (testing with a health professional or offering an HIV self-test), continued care with a health professional, and treatment. The focus is to create the individual’s bond with the health service, monitor, promote health education, and treat HIV and other sexually transmitted infections (STI) [5]. However, these services are not always available or accessible to everyone. For example, TGW have limited access to health services, which include a lower frequency of HIV testing, HIV prevention, and health care in general [6].

Studies show that gender identity discrimination operates as one of the main barriers to accessing health services, including HIV/AIDS services [7–9]. In Brazil, although the National Health Policy for Lesbian, Gay, Bisexual, and Transgender people (NHPLGBT) have ensured the inclusion and non-discrimination of this population in health services, there is still evidence of a violation of TGW’s rights: failure to respect the social name, discrimination in health services, and failure to meet the necessary health need [8, 10, 11]. Thus, gender-based discrimination (GBD) remains one of the main obstacles faced by TGW in accessing health services [12], which can hinder HIV prevention and care and increase the risk to HIV/AIDS [13, 14]. In Brazil, no other studies investigated the association between GBD, HIV testing, and access to medical visits among TGW.

This study aims to investigate the putative association between gender-based discrimination (GBD) and (i) medical visits in the last 12 months and (ii) HIV testing in the last 12 months, among TGW from three large capital cities in Northeast Brazil.

## Methods

This study reports findings from a cross-sectional Biological and Behavioral Surveillance Survey among TGW, conducted in three large capitals of northeastern Brazil. These sites were part of the multicity DIVAS study (National Research Study on Behaviors, Attitudes, Practices, as well as assessment of the Prevalence of HIV, Syphilis and Hepatitis B and C among *Travestis* and Transsexual Women). The DIVAS study was conducted in 12 cities in Brazil from October 2016 to July 2017, aimed to estimate the prevalence of HIV, and other sexually transmitted infections (STI) and monitor risk practices for these infections [15]. DIVAS is one of the largest studies among TGW in a given nation, worldwide.

The study protocol was submitted for review and approved by the Sergio Arouca National School of Public Health (ENSP/FIOCRUZ) Research Ethics Board (CAAE-49359415.9.0000.5240). Written informed consent was asked and obtained from all participants, who could withdraw consent at any stage of the process or skip any questions perceived as too sensitive, too personal, or distressing.

### Study population

TGW (864) were selected from the cities of Salvador (166), Recife (350) and Fortaleza (348) in 2017. They were recruited using respondent-driven sampling (RDS) as a sampling method aiming to obtain a more robust and diverse sample. They were eligible for the study if they self-identified themselves as transgender women, women, or other category different from the male sex designated on their birth certificate; reported spending most of their time at the selected city (living, studying, and/or working there). Each study participant was screened for eligibility prior to enrollment. The inclusion criteria for the current analysis were those as follows: to answer the questionnaire (with the option to skip some questions), to perform STI rapid tests (with the right to opt out), to will to recruit peers for the study, to sign the informed consent form, and to have had at least one sexual intercourse in the past 12 months. The exclusion criteria were: being under 18 years of age and to be under the influence of alcohol or other drugs in the moment of the interview.

### Data collection and sampling

As required by the RDS method, 5 and 10 initial participants in each city − called seeds − were chosen purposively, following formative qualitative research only as an initial stage for recruitment. The formative phase of the study comprised group discussions with local TGW leaders, non-governmental organizations (NGO), potential participants, and researchers. Each seed received three coupons to distribute to other TGW from her social networks. The interviewees recruited by the seeds were defined as the first wave of the study. After participating in the interview, each participant received three additional coupons to distribute to their peers. This process was repeated until the a priori defined sample size was achieved in each site.

RDS requires a system of primary and secondary incentives. The primary incentive was US$10.00 to pay for a light meal and transportation. The secondary incentive was a payment of US$10.00 for each recruited person who participated in the study [16].

Data were collected through interviews with a standardized and previously tested questionnaire, triangulating findings from focus groups and a pilot study. Face-to-face interviews were carried out by thoroughly trained interviewers, in a private space, reserved for this sole purpose.

### Study variables

**Fig. 1:**
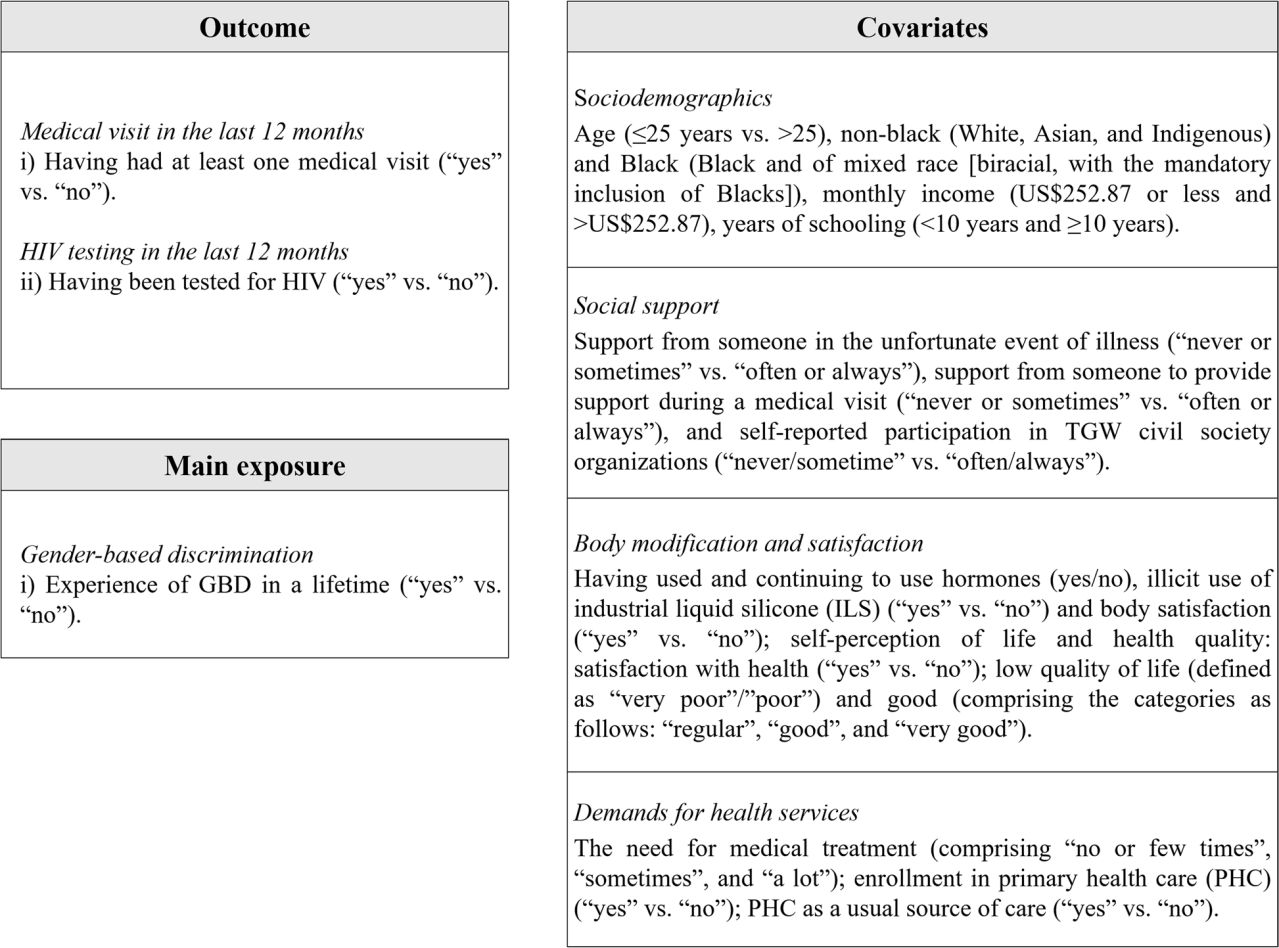
Variables of the study

The variables presented in this study are available in Fig. 1.

### Data analysis

Data analysis took into consideration the complex sampling design of recruitment by RDS [17]. Each one of the three cities was defined as a stratum. In each stratum, the weighting was inversely proportional to the size of each participant's network, totaling the stratum size (RDS-II estimator) [18]. The questions in the questionnaire that measured the network size of each TGW were "How many TGW do you know, by name/nickname and who also know you by your name/ nickname, who live, work or study in your city?” Out of those you mentioned, how many have you met or spoken to personally, by phone or Facebook/WhatsApp within the last 30 days?" The analysis was conducted using the library for complex samples of STATA software version 15 (StataCorp, 2015).

We fitted independent logistic regression models yielding odds ratios (OR) adjusted for potential confounding factors, as well as their respective 95% CI. The variables with a p-value < 0.05 or defined as relevant by their magnitude in the bivariate analysis were included in the adjustments of the final multivariate models.

The bivariate and multivariate equations are presented below. In Eq. 1.0, P is the estimated probability of event occurrence P(Y = 1), represented by the odds of having had medical visits or an HIV test, when the independent variable is ${x}_{1}$, GBD or any other covariate. In Eq. 1.1, P represents the odds of having had medical visits or an HIV test P(Y = 1), when the independent variable ${x}_{1}$ is a GBD, adjusted by the addition of x_t covariates in the model.

1.0 $$P(Y=1)=\frac{{e}^{\left({\beta }_{0}+{\beta }_{1}{x}_{1}\right)}}{1+{e}^{\left({\beta }_{0}+{\beta }_{1}{x}_{1}\right)}}$$

1.1 $$P(Y=1)=\frac{{e}^{({\beta }_{0}+{\beta }_{1}{x}_{1}+{\beta }_{2}{x}_{2}+ \dots +{\beta }_{t}{x}_{t} )}}{1+{e}^{({\beta }_{0}+{\beta }_{1}{x}_{1}+{\beta }_{2}{x}_{2}+ \dots +{\beta }_{t}{x}_{t})}}$$

Percent change in effect of unadjusted and adjusted OR was used to evaluate confounding [19]. The adequacy of the final models was analyzed using the Hosmer–Lemeshow goodness-of-fit test [20].

## Results

Table 1 presents the results describing each one variable analyzed in this study. Out of the total 864 TGW, 772 (87.3%) reported experience of GBD in their lifetime. 547 (67.0%) reported medical visits and 385 (45.8%) HIV testing, both in the past 12 months. Most interviewees were aged > 25 years (50.5%), self-reported black skin color (79.4%), a monthly income > US$252.87 (65.5%), and ≥ 10 years of schooling (54.5%). Most reported that they often or always have the support of someone in case of an illness (65.2%) and for accompany medical visits (57.9%), but few self-reported engagements in TGW civil society organizations (22.9%).

**Table 1:** Distribution of study variables among transgender women in Northeast Brazil, 2017

Variables	n^a^	%^a^
**Outcome**		
**Medical visits in the last 12 months**		
No	271	33.0
Yes	547	67.0
**HIV testing in the last12 months**		
No	458	54.2
Yes	385	45.8
**Main exposure**		
**GBD**		
No	92	12.7
Yes	772	87.3
**Covariates**		
**Age**		
18 to 25	439	49.5
26 and older	425	50.5
**Skin color**		
Non-black	171	20.6
Black	681	79.4
**Monthly income**		
US$252**.**87 or less	293	34.5
> US$252**.**87	571	65.5
**Years of schooling**		
Up to 9 years	378	45.5
10 or more years	473	54.5
**Support in case of illness**		
Never or sometimes	303	34.3
Often or always	553	65.2
**Support to accompany in the medical visits**		
Never or sometimes	358	42.1
Often or always	502	57.9
**Participation in TGW civil society organizations**		
No	638	77.1
Yes	233	22.9
**Hormone use**		
No	417	47.5
Yes	440	52.5
**Illicit use of industrial liquid silicone**		
No	633	87.1
Yes	228	12.9
**Body satisfaction**		
Dissatisfied	396	45.6
Satisfied	462	54.4
**Health satisfaction**		
Dissatisfied	273	29.7
Satisfied	587	70.3
**Self-reported quality of life**		
Poor	62	7.7
Good	796	92.3
**Need for medical treatment**		
No need or very little need	623	71.2
Moderately or very much	230	28.9
**Enrollment in PHC**		
No	254	31.5
Yes	600	68.5
**PHC as a usual source of care**		
No	559	67.0
Yes	304	33.0

*GBD* gender-based discrimination, *TGW* transgender women, *PHC* primary health care

^a^ Weighted by RDS-II estimator

Following the results in Table 1, slightly more than half (52.5%) reported having used and continue to use hormones, and 12.9% reported illicit use of ILS. Most reported being satisfied or very satisfied with their bodies (54.4%), albeit in a smaller proportion than that observed for self-reported satisfaction with their health (70.3%) and self-perception of life quality (92.3%). Most reported enrollment in primary health care facilities (68.5%). However, only one third had primary health care as the usual source of care (33.0%). In addition, most reported no need or little need of a medical treatment (71.2%).

We summarize the results from the bivariate logistic regression analysis (Eq. 1.0) in Tables 2 and 3. At a significance level of 0.05, the independent variables that were significantly associated with medical visits in the last 12 months presented in Table 2 were: GBD (OR 0.37; 95% CI 0.19–0.35); age 26 years and older (OR 1.54; 95% CI 1.03–2.30); monthly income greater than US$252.87 (OR 1.69; 95% CI 1.11–2.56); 10 years or more of schooling (OR 1.54; 95% CI % 1.02–2.32); moderately or very pronounced need of medical treatment (OR 1.99; 95% CI 1.24–3.18); and enrollment in PHC (OR 2.00; 95% CI 1.29- 3.10). In Table 3, for a significance level of 0.05 the independent variables that had a significant association with HIV testing in the last 12 months were the ones as follows: GBD (OR 0.47; 95% CI 0.24–0.91); age 26 years and older (OR 1.76; 95% CI % 1.19–2.60); 10 years or more of schooling (OR 1.64; 95% CI 1.10–2.44); moderately or very pronounced need of medical treatment (OR 2.03; 95% CI 1.32–3.13); illicit use of industrial liquid silicone (OR 1.68; 95% CI 1.08–2.63). and participating in TGW civil society organizations (OR 1.80; 95% CI 1.17–2.77).

**Table 2:** Bivariate analysis of factors associated with medical visits in the last 12 months among TGW in Northeast Brazil, 2017

**Variables**	**Medical visit in the last 12 months**^a^				
	**No (%)**	**Yes (%)**	***p-value***	**OR**	**95%CI**
**Main exposure**					
**GBD**			0.005		
No	16.95	83.05		1.00	-
Yes	35.33	64.67		0.37	0.19–0.75
**Covariates**					
**Age**			0.036		
Up to 25 years	37.89	62.11		1.00	-
26 years and over	28.42	71.58		1.54	1.03–2.30
**Skin color**			0.939		
Non-black	33.55	66.45		1.00	-
Black	33.10	66.90		1.02	0.60–1.73
**Monthly income**			0.014		
US$252**.**87 or less	40.56	59.44		1.00	-
> US$252**.**87	28.80	71.20		1.69	1.11–2.56
**Years of schooling**			0.039		
Up to 9 years of study	37.94	62.06		1.00	-
10 years and over	28.44	71.56		1.54	1.02–2.32
**Support in case of illness**			0.354		
Never or sometimes	36.24	63.76		1.00	-
Often or always	31.86	68.14		1.22	0.80–1.84
**Support to accompany in the medical visits**			0.682		
Never or sometimes	34.22	65.78		1.00	-
Often or always	32.31	67.69		1.09	0.72–1.64
**Health satisfaction**			0.044		
Dissatisfied	26.29	73.71		1.00	-
Satisfied	36.07	63.93		0.63	0.40–0.99
**Need for medical treatment**			0.004		
No need or very little need	37.36	62.64		1.00	-
Moderately or very much	23.08	76.92		1.99	1.24–3.18
**Self-reported quality of life**			0.191		
Poor	24.24	75.76		1.00	-
Good	34.14	75.76		0.62	0.30–1.28
**Hormone use**			0.648		
No	34.12	65.88		1.00	-
Yes	32.03	67.97		1.10	0.73–1.65
**Illicit use of industrial liquid silicone**			0.178		
No	34.82	65.18		1.00	-
Yes	27.99	72.01		1.37	0.86–2.19
**Enrollment in PHC**			0.002		
No	43.23	56.77		1.00	-
Yes	27.60	72.40		2.00	1.29–3.10
**PHC as a usual source of care**			0.173		
No	34.99	65.01		1.00	-
Yes	28.62	71.38		1.34	0.88–2.52
**Participation in TGW civil society organizations**			0.119		
No	34.95	65.05		1.00	-
Yes	26.98	73.02		1.45	0.91–2.33

*GBD* gender-based discrimination, *TGW* transgender women, *PHC* primary health care

^a^ Weighted by RDS-II estimator

**Table 3:** Bivariate analysis of factors associated with HIV testing in the last 12 months among TGW in Northeast Brazil, 2017

Variables	HIV testing in the last 12 months ^a^				
	**No (%)**	**Yes (%)**	***p-value***	**OR**	**95%CI**
**Main exposure**					
**GBD**			0.023		
No	37.91	62.09		1.00	-
Yes	56.52	43.48		0.47	0.24–0.91
**Covariates**					
**Age**			0.004		
Up to 25 years	61.17	38.83		1.00	-
26 years and over	47.23	52.77		1.76	1.19–2.60
**Skin color**			0.623		
Non-black	56.61	43.39		1.00	-
Black	53.57	46.43		1.13	0.69–1.85
**Monthly income**			0.385		
US$252**.**87 or less	57.13	42.87		1.00	-
> US$252**.**87	52.62	47.38		1.20	0.79–1.81
**Years of schooling**			0.015		
Up to 9 years of study	61.11	38.89		1.00	-
10 years and over	48.95	51.05		1.64	1.10–2.44
**Support in case of illness**			0.692		
Never or sometimes	55.39	44.61		1.00	-
Often or always	53.35	46.65		1.09	0.72–1.63
**Support to accompany in the medical visits**			0.876		
Never or sometimes	53.43	46.57		1.00	-
Often or always	54.22	45.78		0.97	0.65–1.44
**Health satisfaction**			0.348		
Dissatisfied	50.49	49.51		1.00	-
Satisfied	55.53	44.47		0.82	0.53–1.25
**Need for medical treatment**			0.001		
No need or very little need	59.36	40.64		1.00	-
Moderately or very much	41.83	58.17		2.03	1.32–3.13
**Self-reported quality of life**			0.383		
Poor	47.83	52.17		1.00	-
Good	55.13	44.87		0.75	0.38–1.45
**Hormone use**			0.898		
No	54.41	45.59		1.00	-
Yes	53.77	46.23		1.02	0.69–1.52
**Illicit use of industrial liquid silicone**			0.021		
No	57.26	42.74		1.00	-
Yes	44.31	55.69		1.68	1.08–2.63
**Enrollment in PHC**			0.204		
No	58.23	41.77		1.00	-
Yes	51.36	48.64		1.32	0.86–2.03
**PHC as a usual source of care**			0.468		
No	52.82	47.18		1.00	-
Yes	56.53	43.47		0.86	0.57–1.29
**Participation in TGW civil society organizations**			0.007		
No	57.46	42.54		1.00	-
Yes	42.88	57.12		1.80	1.17–2.77

*GBD* gender-based discrimination, *TGW* transgender women, *PHC* primary health care

^a^ Weighted by RDS-II estimator

The multivariate analyses (Eq. 1.1) have found statistically significant association (*p* < 0.05) between GBD and the two study outcomes, as shown in Tables 4 and 5. TGW who reported experience of GBD had 71% less likely to attend a medical visit (OR 0.29; 95% CI 0.14–0.63) and 59% less likely to have had an HIV test in the last 12 months when compared to TGW who did not experienced GBD (OR 0.41; 95% CI 0.22–0.78), after adjusting for potential confounders.

**Table 4:** Multivariate analysis of the association between GBD and medical visit in the last 12 months among TGW in Northeast Brazil, 2017

**Model 1**	**OR **^**a**^	***p-value***	**95%CI**	
**GBD**	0.37	0.006	0.19–0.75	
**Model 2**	**OR **^**a**^	***p-value***	**95%CI**	**Percent change in effect**
**GBD**	0.33	0.005	0.15–0.71	11.97
Adjusted for age, monthly income, years of schooling, need for medical treatment, self-reported quality of life, health satisfaction, illicit use of industrial liquid silicone, enrollment in PHC, PHC as a usual source of care, participation in TGW civil society organizations				
**Model 3**	**OR **^**a**^	***p-value***	**95%CI**	**Percent change in effect**
**GBD**	0.30	0.003	0.14–0.66	18.61
Adjusted for age, schooling, need for medical treatment, self-reported quality of life, health satisfaction, illicit use of industrial liquid silicone, enrollment in PHC, PHC as a usual source of care, participation in TGW civil society organizations				
**Model 4**	**OR **^**a**^	***p-value***	**95%CI**	**Percent change in effect**
**GBD**	0.30	0.002	0.14–0.65	19.12
Adjusted for age, schooling, self-reported quality of life, health satisfaction, illicit use of industrial liquid silicone, enrollment in PHC, PHC as a usual source of care, participation in TGW civil society organizations				
**Model 5**	**OR **^**a**^	***p-value***	**95%CI**	**Percent change in effect**
**GBD**	0.30	0.002	0.14–0.65	19.33
Adjusted for age, self-reported quality of life, health satisfaction, illicit use of industrial liquid silicone, enrollment in PHC, PHC as a usual source of care, participation in TGW civil society organizations				
**Model 6**	**OR **^**a**^	***p-value***	**95%CI**	**Percent change in effect**
**GBD**	0.29	0.002	0.14–0.63	21.42
Adjusted for self-reported quality of life, health satisfaction, illicit use of industrial liquid silicone, enrollment in PHC, PHC as a usual source of care, participation in TGW civil society organizations				
**Hosmer–Lemeshow (*p-value*)**	0.979			

*GBD* gender-based discrimination, *TGW* transgender women, *PHC* primary health care

^a^ Weighted by RDS-II estimator

**Table 5:** Multivariate adjustment of the association between GBD and HIV testing in the last 12 months among TGW in Northeast Brazil, 2017

**Model 1**	**OR **^**a**^	***p-value***	**95%CI**	
**GBD**	0.47	0.030	0.24–0.91	
**Model 2**	**OR **^**a**^	***p-value***	**95%CI**	**Percent change in effect**
**GBD**	0.43	0.009	0.22–0.80	9.47
Adjusted for age, years of schooling, need for medical treatment, illicit use of industrial liquid silicone, participation in TGW civil society organizations				
**Model 3**	**OR **^**a**^	***p-value***	**95%CI**	**Percent change in effect**
**GBD**	0.41	0.007	0.22–0.78	12.13
Adjusted for schooling, need for medical treatment, illicit use of industrial liquid silicone, participation in TGW civil society organizations				
**Hosmer–Lemeshow (*p-value*)**	0.995			

*GBD* gender-based discrimination; TGW, transgender women; PHC, primary health care

^a^ weighted by RDS-II estimator

## Discussion

The estimated frequency of medical visits in the last year among TGW was relatively low (67.0%) compared to other studies conducted in the United States (US) of America, a country without a national health system, but rather a patchwork of private and public initiatives, the latter always focused on some populations and delivered on a given catchment area [21, 22]. In San Francisco, an RDS survey of TGW found a 78.0% prevalence of medical visits in the last six months [21]. Data from the US Centers for Disease Control and Prevention (CDC) from 2014 to 2016 indicated a 70.3% prevalence of PHC visits in the last year among TGW [22].

To the best of our knowledge, no studies in Brazil have estimated the prevalence of medical visits in the last year for the TGW population. In general, the production of data on the access of this population to health services comes from qualitative studies [23]. Thus, we still do not have parameters for epidemiological studies that assess such characteristics in Brazil, as there is no question about gender identity in previous studies to make this comparison.

However, the 2013 Brazilian National Health Survey (PNS) investigated this indicator of access to health for the overall adult population [24, 25], and the estimated prevalence was 71.2% for Brazil. These figures were a bit lower for northeastern Brazil (66.3%). This rate is close to the one found in this study for TGW. [25].

Although Brazil has a universal health system (in Portuguese Sistema Único de Saúde- SUS), the services made available by the Unified Health System (SUS) are not always adequate for serving the TGW population. There is still no effective strategy to link these persons to the health system and there are still many discrimination-based barriers to access: discrimination by health professionals and service users, disrespect for the social name and gender identity and failure to meet the necessary health demands. Such barriers can hinder to carry out the medical visit, as well the avoidance of the seeking for health services [10, 26, 27].

Also, medical visits should be a key opportunity to assess TGW’s specific needs. Considering the evidence from epidemiological studies, documenting that TGW face high vulnerability and risk factors for HIV/AIDS [7, 28], the visit should be an ideal moment for the offering and undertaking HIV testing and counseling. The Brazilian Ministry of Health determines a six-monthly HIV testing frequency [29], especially for populations most vulnerable to infection. Thus, the health system should offer and allow effective access to HIV prevention and care technologies.

The estimated prevalence of HIV testing in the last year in this study (45.8%) was less than that found for TGW in Ho Chi Minh, Vietnam (59.3%) in the last year [29], and Cambodia (49.2%) [30] and Pattaya, Thailand (54.7%) [31], in the last 6 months. As in Brazil, both Vietnam, Cambodia and Thailand [4] offer public services for regular HIV testing. Possibly, health services in these countries have more inclusive strategies for these populations compared to the Brazilian situation.

In a review study of 17 countries in Latin America, Silva-Santisteban et al. [32] showed that despite having implemented early HIV prevention and treatment strategies, Brazil was among the countries with the lowest access to testing in the last year for TGW and men who have sex with men (20.3%). Latin American countries with the highest level of access to HIV testing in the past year were Honduras (73.7%), Costa Rica (73.3%), Argentina (58.7%), and Paraguay (56.6%).

Attending medical visits and undergoing HIV tests in the last year indicates access to health services and HIV prevention [33]. However, this study revealed that although more than half of the interviewed TGW reported a medical visit in the last year, less than half underwent HIV testing in the same period. This substantial difference may be explained by some factors. At least, it´s possible to advance plausible hypotheses.

First, not all health services in Brazil that include medical assistance are always suitable for HIV testing. Some problems remain: the unpreparedness of services and health professionals; lack of organization and proper functioning of services, not capable to offer full access to these populations; the current implementation of decentralization strategies for PHC actions has not been effectively implemented; and finally, there is still a lack of communication between the services of PHC and specialized care. [34, 35]. That is, users will not necessarily have access to HIV testing through medical visits. A second issue is HIV/AIDS stigma. The fear of knowing the test result and the negative beliefs often associated with people living with HIV can hinder seeking the test [36].

Specifically, among TGW, besides HIV-related discrimination, GBD can also emerge as an important hurdle. Our study found that GBD was responsible for reducing 71% of medical visits and 59% of HIV testing in the last year, regardless of the other social markers studied in this population. Several other studies have shown that discrimination can be an important barrier to access health services [8, 9, 26, 37–44] and HIV testing [14, 28, 45–55].

In a systematic review study about the barriers faced by TGW in health services, Nascimento, Sousa, and Barros [12] showed that stigma and discrimination are still relevant as one of the main barriers faced by these populations. Found in several social contexts, including health services, GBD prevents them from obtaining adequate care [53, 54].

Concomitantly, by affecting access to health services, discrimination also affects access to HIV testing services. In a study with a RDS of TGW from Fortaleza, Brazil, Pinheiro Júnior et al. [14] revealed that being discriminated against increases almost fourfold the resistance to HIV testing in a lifetime. Logie et al. [16] showed that the probability of having an HIV test in a lifetime decreases with increased HIV-related stigma and that, possibly, stigma is also found in testing services.

A systematic review study on the stigma endured by TGW, Magno et al. [7] showed that stigmatization is an important element that makes this population vulnerable to HIV/AIDS, mainly through social exclusion and violence. These authors also argued that discrimination develops as a factor that hinders access to health services, causing barriers to access tests and other services and making TGW vulnerable to HIV/AIDS.

Profound changes are still required to denaturalize and disassemble GDB in society and the health system. Rocon et al. [27] argue that it is necessary to reassess the production of care by health professionals and the inclusion of the transgender population in a participatory way. Public health policies should sufficiently structure the entire service network to offer equitable access and foster effective social control [55].

This study has some limitations. Due to the difficulty of defining the sample size of an RDS study by the classic method at the time of conducting this research, we used samples of size defined a priori by the Brazilian Ministry of Health. The RDS method design prevents the generalization of our data. A selection bias may have occurred as it represents a non-probabilistic sample, and recruits invited their peers [16]. However, it does not prevent us from providing important information about the social network established. Several other studies have used this same sampling method to study TGW [14, 56–59].

Another limitation of this study is the questionnaire. It was not particularly suitable for analyzing the outcomes since it was originally designed for a comprehensive assessment, not necessarily focusing on our specific questions. For this reason, we were unable to evaluate the putative role of other confounding variables. The self-perceived discrimination variable may not capture internalized or imperceptible experiences and may underestimate our results [60]. Notwithstanding this, it also did not stop revealing statistically significant data since the sampling power was adequate in this case.

## Conclusion

The study showed that the prevalence of medical visits in the last 12 months did not match the expected number and respective proportions of HIV testing in the last 12 months among TGW, which points to the loss of HIV testing opportunities since this is a population in a context of high vulnerability and risk of HIV infection. We recommend that medical visits in Brazil carry out at least the investigation of previous HIV testing and, based on anamnesis data, indicate at least one test per year, as it is already done in other countries.

Also, we observed that GBD were reported in most of the TGW, and reduced the likelihood of medical visits and HIV testing in the last 12 months. This data reinforces the role of discrimination as an important obstacle to health services and HIV testing. Thus, it is necessary to create and strengthen health policies and laws to protect TGW against discrimination, as well as to increase the equitable access of this population to public health services and HIV prevention and testing technologies.

## Data Availability

The dataset analyzed that underlie the results reported in this article will be publicly available in the HAVARD Dataverse Repository immediately following publication.

## Abbreviations

GBD: Gender-Based Discrimination
HIV: Human Immunodeficiency Virus
NGO: Non-Governmental Organizations
NHPLGBT: National Health Policy for Lesbian, Gay, Bisexual, and Transgender people
RDS: Respondent-Driven Sampling
STI: Sexually Transmitted Infections
WHO: World Health Organization
